# Preserving misconceptions or a call for action? – A hermeneutic re-reading of the Nativity story

**DOI:** 10.3402/gha.v8.30386

**Published:** 2015-12-24

**Authors:** Mats Målqvist

**Affiliations:** 1 International Maternal and Child Health, Department of Women's and Children's Health, Uppsala University, Uppsala, Sweden; 2 Church of Sweden, Uppsala, Sweden

**Keywords:** structural determinants, religion, maternal health, child health, health determinants

## Abstract

**Background:**

Behaviour is guided by perceptions and traditions. As such, understanding culture and religion is important in order to understand healthcare behaviour. Religious perceptions shape a person's understanding of the world and are maintained through texts and tradition. One such important religious text in relation to sexual and reproductive health is the Nativity story. This account of the conception and birth of Jesus is well known in the Christian cultural sphere and beyond, and it has for generations shaped perceptions of childbirth.

**Methods:**

This paper attempts a re-reading of the Nativity story using a hermeneutic approach.

**Results and Conclusion:**

This reveals a dual understanding of the Nativity, not just as an account of immaculate transcendence and a rosy Christmas tale, but as a source of identification for pregnant women and mothers and a call to action for improved maternal and child healthcare.

## Introduction

Ideas shape our minds and our minds shape our behaviour (1). Concepts of what is appropriate or right guide the way we do things (2). Thus, tradition and ‘how we have always done things’ can become great barriers to the introduction of evidence-based practice (3, 4). This tendency is particularly evident in maternal and child healthcare. The family, and reproduction, are central concepts around which we arrange our lives, and the surrounding structures are well guarded by tradition. Sexual relations, marriage, childbearing, birth, and the rearing and place of children are, in all cultures, heavily regulated to guide our relations and behaviour (5). The cultural rules and traditions surrounding maternal and child health have been developed throughout history to protect humankind from internal and external threats. For example, the 1-month seclusion after delivery for mother and newborn, which is so common in many cultures, is aimed at protecting the neonate from infection. Today, however, we have new knowledge, and antibiotics to cure early childhood infections as well as home visits by healthcare workers to detect and act upon warning signs of infection. All of these interventions help to save a large proportion of the newborns who would otherwise die (6). Cultural traditions and misconceptions can thus stand in the way of recent evidence that challenges how things are done, and they can be an obstacle to implementing positive behavioural change (7, 8). Maternal and child health practitioners and researchers therefore need to address not only the medical side of the challenge, but also to obtain a deeper understanding of the cultural and contextual factors guiding end receivers’ behaviour.

Religion plays a vital part in bringing sense and meaning to reality (1). All humans, from atheists to fundamentalist Buddhists, are guided in their behaviour by their perceptions and explanatory models of reality (1). There is also a strong link between religion and health, an aspect that is many times neglected in healthcare research and delivery (9). Major life course events such as birth, becoming a parent, and raising a child are all surrounded by rules and regulations derived from our religious and cultural thought patterns (7, 8). To preserve these thought constructs, most religions have texts, sacred or traditional, to outline and encompass their messages and ideas. Even in oral traditions, repetition and preservation of the inherited storyline are essential elements (2). The text is a sustaining force, a point of reference for religion's power of explanation. As such, religious texts create normalcy and can consequently be an important key to understanding behaviour (2). One such text in the Christian tradition is the Nativity story, narrating the birth of Jesus. In the Christian tradition the Nativity story serves as the statement of Mary's virgin birth and Jesus’ subsequently immaculate nature, proclaiming Jesus as the Son of God (10). As such, the Nativity is central to the theological message of Christianity. However, the story can also be, and has been, read as a story of a regular birth, stating and advocating for the humanity of Jesus, showing how the Son of God is also subject to normal human hardship (10). In this capacity the Nativity story has become a popular folk tale, shaping perceptions of pregnancy and childbirth. Given the spread of this account of childbirth and the authority it has as a religious text, it is important to critically examine the text in the light of what we know and advocate for today in global maternal and child health. The aim of this paper is to apply a hermeneutic approach to the Nativity story, as it is portrayed in the Gospel of Luke.

## Methods

### Hermeneutics – contextual interpretation

**Fig. 1:**
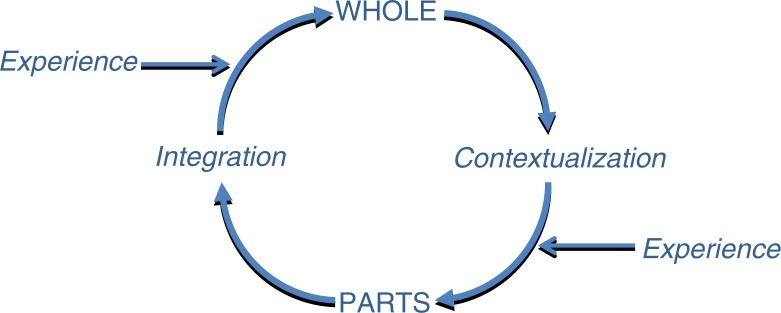
The hermeneutic circle of interpretation.

*Hermeneutics* can be described as the interpretation of text; it is a research methodology that departs from what is currently known. There are many schools and adaptations of hermeneutics, but basically hermeneutics set up rules for interpretation. By various means hermeneutic methods strive to create a deeper understanding and gain new insights by moving from the whole to the details and back to the whole, with input of experience and contextual interpretations along the way (Fig. 1) (11). The pre-understanding of a text, phenomenon, or cultural expression therefore has an important role in hermeneutics, as there is no objective starting point in relation to a text. The pre-understanding leads to understanding, which in turn becomes the new pre-understanding, and the hermeneutic circle is thus closed. Experience and new knowledge have an important role to feed into the hermeneutic circle and thereby fuel the process of a deeper and sometimes different understanding (Fig. 1) (11).

## Results

### A religious success story

The Nativity story is many times perceived as something of a religious success story, an account of an immaculate birth, with angels singing and the whole of creation celebrating the birth of its saviour (10). The account from the Gospel of Luke is read in churches and homes all over the world at Christmastime. Mary, the mother of Jesus, is perceived as a model of virtue, with a serving spirit, being the chosen one for the glorious mission of carrying the Son of God in her womb (12). In many churches, especially within Catholicism, the Virgin Mary has an uplifted and celebrated position, not far removed from Jesus himself. This elevated status comes from an interpretation of the Nativity story as something of grandeur, with Mary as its main heroine (12). In this way the pregnancy and childbirth of Mary becomes a role model account, not only shaping normality but also depicting the perfect.

### An unwanted pregnancy

The hermeneutic approach now directs us to go from the whole, as described in the previous paragraph, to look more closely at the details (Fig. 1). This is done with the contextual addition of today's global maternal and child health. The first thing that can be noted is that the story begins with an unwanted pregnancy, a major challenge in maternal and child health. The message the angel delivers in Luke 1:31 is not only unexpected, but must in today's terms also be considered an unwanted pregnancy. Mary however does not protest by claiming the right to her own body, but succumbs to patriarchal authority (Luke 1:38). Angels in the Bible are usually men, and the response of Mary, to internalize the unexpected news of pregnancy and ‘hide it in her heart’, is a common strategy among many young women who cannot exercise the right over their own bodies. The unwanted pregnancy is not only stressful because of the biological changes that come with it, but also because of the high level of stigma associated with it. To be found pregnant out of wedlock was considered a crime in the Jewish culture of the time, and the penalty was death by stoning. Joseph, who was engaged to Mary, wanted to avoid the shame of having his betrothed being impregnated by another man. According to the gospel it took divine intervention for him to change his mind (Matthew 1:20). Mary subsequently decides to leave her hometown and travel to her older relative, Elizabeth (Luke 1:39–45). Most likely this was to conceal the pregnancy and avoid the associated stigma until it was undeniable. Mary stayed away for the first full trimester, thus avoiding pressure by family members and society at large to have an abortion, which may very well have been an option at the time. The situation many women find themselves in today is very similar (13). It is quite possible that Mary planned to stay at her relative's house until the baby was born. This strategy remains quite common in unwanted pregnancies today and creates an unsafe environment for the newborn even before birth.

### An unsafe delivery

The rest of the pregnancy seems to have been without complications and the next part of the Nativity story describes the delivery. It is preceded by a long and arduous journey, from Nazareth to Bethlehem (Luke 2:4–5), a trip of about 120 kilometres. Lack of rest and hard physical exertion are known risk factors for preterm birth. This might also be the reason for the poor birth preparations made by the two parents, resulting in Mary having to give birth in a stable (Luke 2:7). There is furthermore no account of any assistance at the birth, except maybe from Joseph. The delivery can hardly be considered to have been hygienic as the newborn was placed in a feeding trough for animals (Luke 2:7). Whether Mary initiated breastfeeding within one hour or not, as is the standard recommendation today, is not mentioned in the text, but it seems that skin-to-skin care was not practised. The gospel mentions explicitly that the newborn was ‘wrapped in cloths’ (Luke 2:12), indicating that this was the preferred method of the day. The account of Jesus’ birth thus contains many examples of unfavourable birth conditions, as outlined in Table 1.

**Table 1:** Observed reproductive health problems in the Nativity story

	Reproductive health problems observed		Possible and observed results	
An unwanted pregnancy	•	Stigma of becoming pregnant out of wedlock	•	Concealment of pregnancy, lack of antenatal care
	•	Risk of abortion	•	Stress in mother
An unsafe delivery	•	Hard physical strain late in pregnancy	•	Risk of preterm birth
	•	Poor birth preparations	•	Risk of stillbirth
	•	No skilled attendance at birth	•	Risk of maternal death
	•	Poor hygiene at delivery	•	Risk of neonatal death
	•	Lack of skin-to-skin care	

### A problem catalogue

**Fig. 2:**
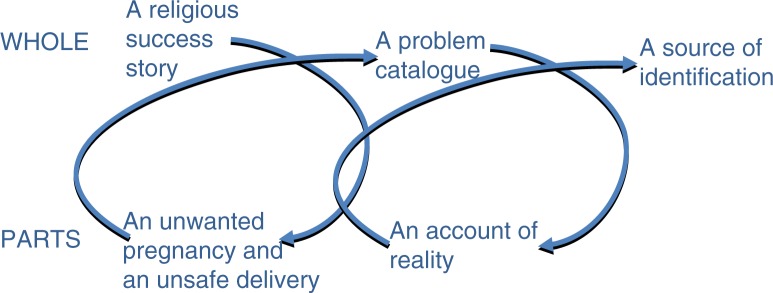
Hermeneutic interpretation of the Nativity story.

When we now continue in the hermeneutic circle we find that the Nativity story is far from a success story (Fig. 2). It is filled with public health risks and the picture that emerges is one of vulnerability and distress. Societal rules and regulations surrounding reproduction often cause problems when a pregnancy does not follow the accepted pattern, resulting in stigma and shame (5). Mary is forced to choose to leave her home or to suffer disdain from her neighbours and even possible death for her and/or her unborn child. In addition, the delivery as a whole is filled with hardship and challenges and serves as more or less a catalogue of things to avoid for a healthy delivery. Lack of birth preparations, skilled assistance at birth, and proper postnatal care create far less than the perfect delivery situation. We can hence conclude that one lap in the hermeneutic circle leaves us with a completely different perception of the Nativity story.

### An account of reality

However, let us take another turn in the analysis and look at the parts in the light of today's global maternal and child health situation (Fig. 2). Millions of women still deliver without skilled assistance at birth and there is a great challenge in the implementation of high quality postnatal care (14). Almost three million newborns die every year during the first 4 weeks of life (14), despite existing evidence that simple interventions such as skin-to-skin care, early initiation of breast feeding, kangaroo mother care, and neonatal resuscitation could save the larger part of these newborn babies (6, 15, 16). The maternal and neonatal health problems identified in the Nativity story are consequently not unlike the current situation. At the time of Jesus’ birth these conditions would have been commonplace. In addition, the issue of unwanted pregnancies, which is a major public health challenge given that the unmet need for family planning is still high and there is little or no access to safe abortion methods, was probably a frequent problem at the time of Mary.

### A source of identification

The picture that emerges after a second round in the hermeneutic circle is a rather blunt and straightforward description of the vulnerable situation of pregnancy and childbirth still experienced by many women today. Far from being a rosy account with heavenly hosts and adoring shepherds, the Nativity story depicts a number of problems addressed in the continuum of care. It thus becomes a source of identification rather than a testimony of immaculate transcendence. Maybe it is there the strength of the story lies, in the tension between reality and divinity? Mary has become an almost divine figure, worshipped by millions the world over, while at the same time being subject to many of the same conditions still faced by so many women today.

## Discussion

### A preserving force or a call to action?

Religious texts shape our perceptions of the world, not only in relation to the divine, but also by making sense of reality through establishing standards and norms for behaviour (2). We have shown that a very well-known and influential religious text like the Nativity story can be interpreted in different ways depending on which perspective is applied. To overcome barriers to good health it is important to get a deeper understanding of underlying ideas and thought patterns that shape behaviour (7, 9). Ideas of normality can many times prevent patients from seeking adequate care and may influence the level of empowerment in individuals. What is considered normal can be a source of resilience, but also a barrier for the acquisition or implementation of new knowledge. As a modelling benchmark, the Nativity story becomes a preserving force, maintaining misconceptions and harmful behaviours in relation to pregnancy and childbirth. On the other hand, the Nativity story supplies a description of reality with which many women can identify and thereby invokes a feeling of community that can spur action. To see that you are not alone, that even the Mother of God suffered suboptimal pregnancy and delivery care, subject to restricting cultural norms, can be a motivation to demand change. As such, the Nativity story becomes a call to action!
